# Non-Pharmacologic Interventions for Caregivers of People With Dementia in Latin America: A Review

**DOI:** 10.1093/geroni/igaa057.881

**Published:** 2020-12-16

**Authors:** Jose Aravena, Jean Gajardo, Laura Gitlin

**Affiliations:** 1 School of Public Health, Yale University, New Haven, Connecticut, United States; 2 Universidad San Sebastián, Santiago, Chile; 3 Drexel University, Philadelphia, Pennsylvania, United States

## Abstract

In a scenario of increasing longevity and social inequalities, Latin-America is an important contributor to the worldwide dementia burden. Caregivers’ health is fundamental to maintain the person with dementia quality of life. However, caregiving is a culturally sensible role that requires tailored solutions. The aim is to synthesize the evidence about non-pharmacologic interventions targeted to caregivers of people with dementia in Latin-American contexts. A comprehensive review of interventions in caregivers and persons with dementia in Latin-American countries was conducted using MEDLINE, Embase, PsycINFO, and Scopus with studies published until January 27th, 2020. Randomized clinical trials of non-pharmacologic interventions targeted to caregivers of people with dementia or dyads where included. Qualitative synthesis of the evidence was presented and analyzed. Overall, 9 pilot RCT were included for the final analysis (6 Brazil, 1 Colombia, 1 Mexico, 1 Perú). The biggest study recruited 69 caregivers and the smallest 13 dyads, with follow-up range of 3-6 months. 5 control groups received at least some other non-standard care type of intervention. 8 were targeted exclusively to caregivers (4 group intervention, 3 individual, and 1 combined) and 1 multicomponent intervention. Most frequent measured outcomes were caregiver’s burden, anxiety, depressive symptoms, and quality of life, and person with dementia neuropsychiatric symptoms. Individual interventions report better results in caregiver parameters such as burden and depressive symptoms and person with dementia neuropsychiatric symptoms. Group interventions presented mixed results. Nevertheless, the quality of evidence was low. There is a critical need to study interventions for caregivers in Latin-American contexts.

